# Attention training technique delivered in groups as treatment for anxiety and depression in patients with coronary heart disease: study protocol for a waiting-list randomized controlled trial

**DOI:** 10.3389/fpsyg.2023.1226539

**Published:** 2023-09-18

**Authors:** Toril Dammen, Kristoffer Tunheim, John Munkhaugen, Ole Klungsøyr, Costas Papageorgiou

**Affiliations:** ^1^Department of Research and Innovation, Division of Mental Health and Addiction, Oslo University Hospital, Oslo, Norway; ^2^Institute of Clinical Medicine, Faculty of Medicine, University of Oslo, Oslo, Norway; ^3^Department of Medicine, Drammen Hospital, Vestre Viken Trust, Drammen, Norway; ^4^Department of Behavioural Medicine, Institute of Basic Medical Sciences, University of Oslo, Oslo, Norway; ^5^Department of Psychology, University of Oslo, Oslo, Norway

**Keywords:** attention training technique, anxiety, depression, rumination, worry, psychological intervention, coronary heart disease

## Abstract

**Introduction:**

Clinically significant symptoms of depression and anxiety in coronary heart disease (CHD) patients are common and associated with adverse outcomes. Psychological treatments have shown limited effectiveness and more effective treatments have been requested. Attention training technique (ATT), a component of metacognitive therapy, can potentially be effective as a stand-alone treatment for anxiety and depression. In an open study, ATT delivered face-to-face in a group format was feasible and potentially effective for improving depression and anxiety symptoms in CHD patients. The next progressive step is to test the effectiveness of ATT in a randomized controlled trial. This paper describes the methodology of this trial.

**Methods:**

ATT-CHD is a randomized wait-list (WL) controlled study. Eligible CHD patients from two hospitals with Hospital Anxiety and Depression Scale (HADS)-Anxiety and/or HADS-Depression subscales scores ≥8 will be randomized into ATT (*n* = 32) or WL control (*n* = 32). After 6–8 weeks, WL patients will be allocated to ATT. Participants will be evaluated pre-, mid- and post-treatment, and at 6-months follow-up using changes in HADS as primary outcome. Secondary outcomes will be changes in psychiatric disorders, rumination, worry, type D-personality, metacognitions, insomnia, quality of life, and C-Reactive protein (CRP).

**Discussion:**

To our knowledge, this will be the first WL-controlled randomized study testing the effectiveness of group-based ATT as treatment of symptoms of anxiety and depression in CHD patients. It will also explore correlations between changes in psychological distress and CRP. A qualitative analysis will reveal patients’ experience with ATT including processes that may facilitate or serve as barriers to effectiveness. Recruitment into the study commenced in December 2022.

**Ethics and dissemination:**

The Regional Committees for Medical and Health Research Ethics (REK), Norway has granted approval for the study (ID 52002). The trial results will be published in peer-reviewed journals. According to Norwegian legislation, the Norwegian Data Protection Authority, and the Committee of Ethics, we are not allowed to share original study data publicly.

**Clinical trial registration:**

ClinicalTrials.gov, identifier NCT05621408 pre-inclusion. There were no significant changes of methods or outcomes after study start.

## 1. Introduction

Coronary heart disease (CHD) is one of the leading causes of death worldwide (Nowbar et al., 2019). Psychological distress such as significant symptoms of depression and anxiety are common in CHD patients (30–40%) (Reid et al., 2013) and commonly occur both immediate and longer periods of time after an acute cardiac event (Palacios et al., 2016). In an observational study from Norway, we reported prevalence rates of depression and anxiety of 14.7 and 21.1%, respectively, as assessed by the Hospital Anxiety and Depression Scale (HADS), mean 16.7 months after a CHD event (Frøjd et al., 2022b).

Patients with CHD have increased mortality risk with depression or anxiety alone being associated with up to two-fold increased risk of cardiovascular events and mortality (van Melle et al., 2004; Watkins et al., 2013). Depression and anxiety commonly co-occur and in those with both conditions, the prognosis is particularly poor with three-times increased risk of mortality (Watkins et al., 2013). Furthermore, associations between depression and anxiety have been reported for higher rates of cardiovascular risk factors, such as smoking and low physical activity, lower treatment adherence and higher levels of markers of immune function (Munkhaugen et al., 2018). The latter risk factor, more specific C-Reactive Protein (CRP), is an independent risk factor for recurrent cardiovascular events in CHD patients (Ridker et al., 2017). In addition, anxiety and depression symptoms are linked to poorer quality of life, higher rates of hospital admissions and work retention (Holdgaard et al., 2023).

Consequently, the most recent European Society of Cardiology guidelines on CVD prevention recommends assessments of psychological needs, including screening for anxiety and depression (Visseren et al., 2021). The importance of assessments of psychological distress in CHD and further intervention have been strongly emphasized (Thompson and Pedersen, 2023). However, a recent Cochrane review of studies on available drug and psychological treatments have showed only small effects on such symptoms and quality of life, and no effect on cardiac prognosis (Richards et al., 2017). Indeed, Richards et al. (2017) found no specific psychological intervention recommendable, and only small improvements in the interventions tested. More recently, Wells and colleagues reported metacognitive therapy (MCT) as a promising group delivered treatment in reducing psychological distress and improving quality of life in cardiac rehabilitation patients an evaluated their MCT interventions in cardiac patients (Wells et al., 2021, 2022a,b). Another recent multicenter RCT on cognitive-behavior therapy (CBT) in cardiac rehabilitation (CR) patients found that supplementing CBT to CR could improve anxiety, depression, quality of life as well as cardiac readmissions (Holdgaard et al., 2023). These effects were maintained at 6-months follow-up.

The attention training technique (ATT) is a component of metacognitive therapy, a treatment approach grounded in the metacognitive model (MCM) of psychological disorder (Wells, 2009). The MCM is based on the principle that particular transdiagnostic processes that represent biases in self-regulation of thinking are major contributors to psychological disorders. A central mechanism is sustained processing of threat and persistent negative ideation-focused coping typified by worry and rumination; a reactive style that is collectively termed the cognitive-attention syndrome or CAS (Wells and Matthews, 1994). The CAS is thought to emerge from biases in metacognition that includes the person’s knowledge and internal programs for regulating thinking and attention (Wells, 2009). Techniques such as the attention training are designed to facilitate the modification of such metacognitions and enhance attention flexibility such that an individual can choose not to engage in the CAS when negative cognitions occur. There is firm evidence that MCT is effective in treating depression and anxiety disorders (Normann and Morina, 2018), and potentially more effective than traditional CBT (Normann et al., 2014; Nordahl et al., 2018) and that individual techniques of MCT such as ATT, can be effective in their own right (e.g., Fergus and Bardeen, 2016).

ATT consists of an auditory attention task that takes about 12 min to practice (Wells, 2009). Consistent with predictions of the S-REF model, ATT has been found to increase attentional flexibility (Barth et al., 2019) and be effective for a range of different psychiatric disorders including symptoms of depression and anxiety as a stand-alone treatment (Fergus and Bardeen, 2016). Recently, RCTs have reported ATT delivered in group formats to be effective in reducing symptoms of anxiety and depression among a student population (Haukaas et al., 2018). There is also preliminary evidence from an open study that ATT is feasible and potentially effective in treating symptoms of anxiety and depression in CHD patients (Dammen et al., 2022).

In addition to improving symptoms of anxiety and depression, because of its mechanism of action, ATT may improve biological markers associated with CHD. Several reviews of the effects of psychological interventions on systemic levels of inflammatory biomarkers have concluded that psychological interventions appear efficacious in reducing CRP, for example, Savin et al. (2022) who reviewed studies of CBT for insomnia and (O'Toole et al., 2018) who included a broader range of therapies. However, no study has measured the effect of ATT on CRP-levels and the associations between distress symptom change and changes in CRP. Such knowledge might provide further evidence of the link between psychological distress and poor cardiac prognosis and has been required (Richards et al., 2017).

In summary, ATT is an effective approach in treating anxiety and depression in clinical and non-clinical populations and delivered individually and in group formats. ATT has potentially many advantages for its use in the CHD patient group. It is derived from a transdiagnostic model of emotional disorder, hence allowing disparate presentations to be addressed with the same core treatment approach. ATT aims to address underlying mechanisms which could represent obstacles to recovery in traditional therapies. It is easier to implement than traditional therapies because it does not require dealing with aspects of life or symptoms that are difficult for patients to confront during therapy. ATT does not require the same level of training and resources to administer as traditional psychotherapies. Instead, ATT consists of exercises that could be administered by trained general practitioners or cardiac nurses and it does not necessarily require a mental health therapist with training in specific therapies. This makes it particularly well suited to CHD patients who often present with mixed anxiety and depression. Finally, in a recent open study, we found ATT delivered face-to-face in a 6- session group format to be feasible and potentially effective for improving depression and anxiety symptoms in CHD patients (Dammen et al., 2022), which holds considerable appeal for use in increasingly busy cardiac follow-up clinics. The next progressive step is to test the effectiveness of ATT in a randomized controlled clinical trial. This study will investigate the effectiveness of ATT compared to a wait list control condition (WL). Those in the WL will also receive ATT after 6–8 weeks (“delayed ATT”). Changes from post treatment to 6 months after treatment will be reported for all treated patients and comparisons will be made at 6 months follow up for those who received “immediate ATT” and “delayed ATT.”

Furthermore, a qualitative study will be “nested” within this RCT. Including a qualitative approach will allow a greater focus on the perspective of the users. To our knowledge, no previous studies have explored how patients experience aspects of ATT delivered in a group format.

### 1.1. Objectives

The overall aim of this study is to provide evidence for the effectiveness of ATT delivered in a group format in CHD outpatients.

The primary objective is to test the effectiveness of ATT delivered in a group format in alleviating symptoms of anxiety and depression in CHD outpatients who experience significant anxiety and depression following a CHD event.

Secondary objectives are (i) to evaluate the impact of ATT on secondary outcomes such as psychiatric disorders, rumination, worry, type D-personality, insomnia, quality of life and metacognitions (ii) explore whether the biological marker CRP that may link psychological factors to cardiac prognosis is correlated with treatment outcome, and (iii) provide qualitative data on the patients experience with ATT including processes that may facilitate or serve as barriers to effectiveness in order to help further improvement in effective delivery and implementation of ATT in cardiac practice.

## 2. Methods and analysis

### 2.1. Study design

**Figure 1:**
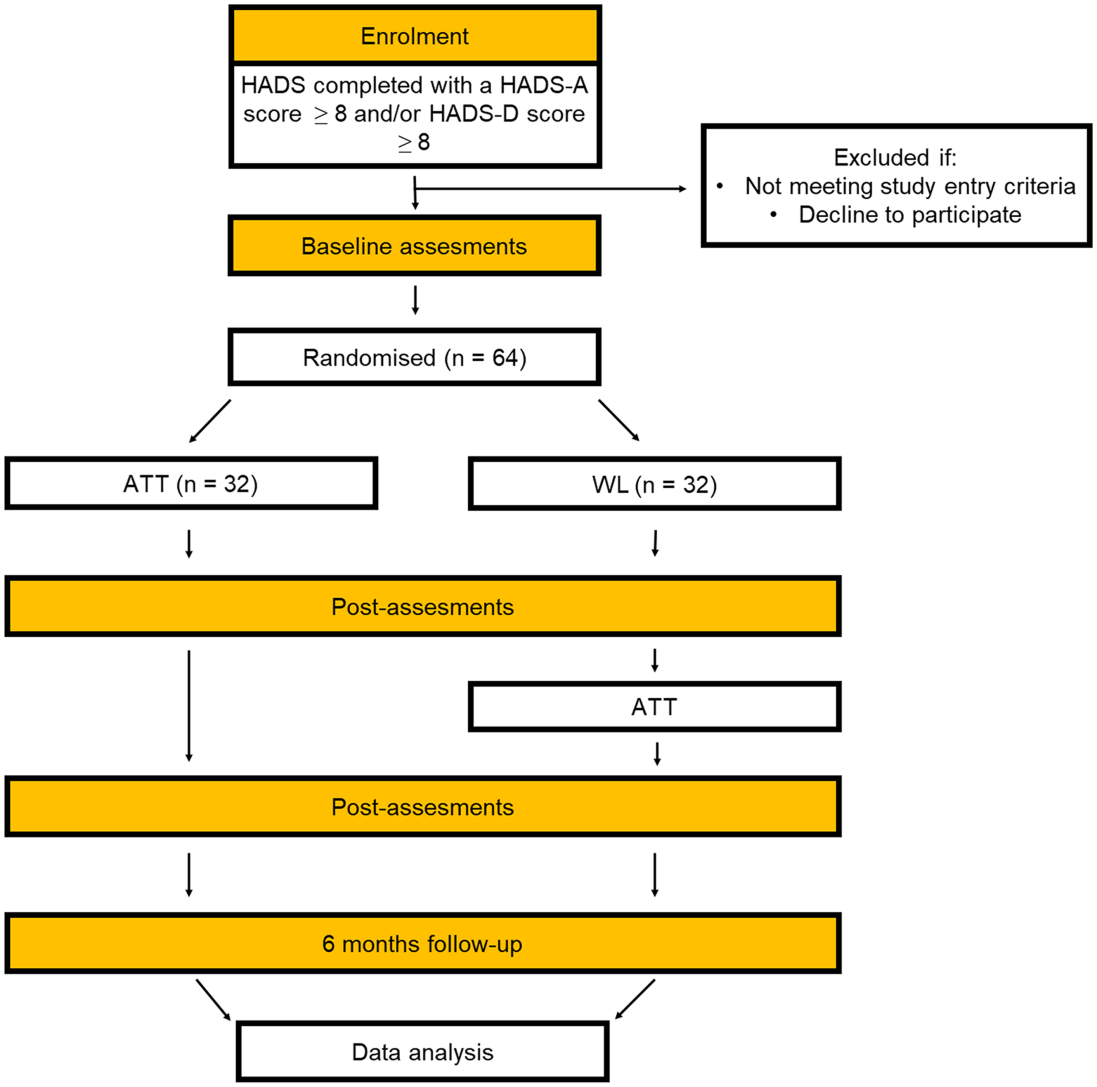
CONSORT flow diagram.

ATT-CHD is a multi-center single-blind waiting list randomized controlled trial with 6 months’ follow-up comparing ATT delivered in a group format with a wait-list control condition. Wait-list control condition is the chosen comparison arm because there is currently no benchmark treatment for distress in this group, existing treatments have limited effects, and this is the first test of group ATT in CHD patients. There are also qualitative evaluations embedded within the trial. A trial flow diagram is shown in Figure 1. An overview of assessments is provided in Table 1.

**Table 1:** Assessments (measurement times are marked with a cross).

Week	SCRE	Pre	w1	w2	w3	w4	w5	w6	Post	Pre	w1	w2	w3	w4	w5	w6	Post	6 mo. FU
Inclusion/exclusion criteria																		
Informed consent																		
Socio-demographics																		
HADS																		
RRS																		
PBRS/NBRS																		
MCQ-30																		
DS-14																		
BIS																		
PSWQ																		
SF12																		
SCID-I/II																		
HsCRP																		

SCID, Structured Clinical Interview for DSM; RRS, Ruminative Response Scale; PBRS, Positive Belief About Rumination Scale; NBRS, Negative Belief About Rumination Scale; MCQ-30, Metacognitive Questionnaire−30; DS14, Type D Questionnaire; BIS, Bergen Insomnia Scale; DMQ, PWSQ, Penn State Worry Questionnaire; SF12, Short-Form−12; HADS, Hospital Anxiety and Depression Scale; hsCRP, high-sensitivity C-Reactive Protein; w, week; mo, month.

### 2.2. Study setting

The study will take place at two large general hospitals (Drammen and Vestfold) in Norway. The study is granted approval by the South-Eastern Norway Regional Health Authority (Helse Sør-Øst RHF/Grant number 2019125).

### 2.3. Study population

The study population will include patients previously hospitalized with myocardial infarction and/or angiography-verified atherosclerosis. The hospitals catchment area corresponds to 7.4 percent of the Norwegian population. It is representative of Norwegian geography, age distribution and morbidity (Kristiansen et al., 2021).

### 2.4. Eligibility criteria

Patients will be asked to participate in the study if they meet the following inclusion criteria:

1. Previously hospitalized with myocardial infarction and/or angiography-verified atherosclerosis.
2. A score of ≥8 on the HADS-anxiety and/or depression subscales.
3. Age ≥ 18 to ≤65 years.
4. Ability to understand and speak the Norwegian language.
5. Signed informed consent.

Patients will be excluded if they meet any of the following criteria:

1. Current or past neurological illness, traumatic brain injury.
2. Current alcohol and/or substance dependency disorders.
3. Psychotic disorders, bipolar disorders, developmental disorders or learning disabilities.
4. Cognitive impairment which precludes informed consent/ability to participate.
5. Acute suicidality.
6. Life-expectancy less than 12 months.
7. Concurrent psychological intervention for emotional distress.
8. Antidepressant or anxiolytic medication initiated or changed during the past 8 weeks.

### 2.5. Recruitment and allocation

Consecutive patients hospitalized with a CHD event during the past years (2011–2022) at the department of cardiology at Drammen hospital or Vestfold hospital will be assessed for eligibility. All potentially eligible patients will be selected from hospital discharge lists by searching chronologically after last admission for the index event during the past years (2011–2022) prior to study inclusion. A specially-trained research cardiac nurse will screen the hospital records. All eligible patients (*N*⁓1,500) will be mailed a comprehensive self-report questionnaire as part of a follow up study addressing psychological distress. The responses will be screened by a research nurse and those who score ≥ 8 for HADS-A and/or HADS-D will be mailed information about the ATT-CHD study and consent to be contacted for further information and evaluation if they return a signed informed consent form together with a recent score ≥ 8 on either of the HADS subscales. Those who meet our eligibility criteria will be offered a face-to-face evaluation, and information about the study if eligible. Those who consent to participate will undergo diagnostic interviews as well as self-report assessments before being randomized and allocated to either ATT immediately or wait-list control. Randomization will be performed by a study statistician blinded to the results of the evaluations. An online software tool is used for randomization with a block- and list-size of 8–12 persons (Sealed Envelope Ltd, 2022. Create a blocked randomization list^1^). The randomization program will be kept in a secured, logged hard disk. Before randomization, baseline data will be collected (Table 1).

### 2.6. Intervention: ATT provided in a group format

ATT was developed by Wells (1990) with the aim of facilitating flexible control of attention. The protocol has been tested in anxiety disorders and in depression (e.g., Papageorgiou and Wells, 2000). The intervention will follow the protocol as described in Wells (2009) treatment manual. The participants will be introduced to the rationale for ATT and explanation of internal and external focus of attention. A rationale will further be given for how ATT may aid in shifting focus of attention and subsequently assisting in overcoming psychological distress. Consistent with Wells (2009), ATT was embedded within meta-level discourse during each session.

The treatment will consist of six weekly group sessions of 60–90 min duration. The number of sessions was based on the results of our feasibility study (Dammen et al., 2022) that is also in line with the number of sessions applied in group MCT study in cardiac patients by Wells et al. (2020). The first session will focus on an explanation of psychological distress in the context of CHD, self-focus of attention, rationale, and practice of ATT as a method of counteracting excessive self-focus. Participants will be informed that they can learn to let go of worry and rumination (overthinking) with the help of ATT. In line with the treatment manual (Wells, 2009), after the initial session, the structure of the sessions will be as follows: review of last session and homework assignment, ATT practice with scoring of self-focus of attention before and after, homework with ATT practice.

ATT is an auditory attention task that takes about 12 min to practice and will be provided to participants as CD or mp3/4 files. The ATT version is a Norwegian translation of the original Wells (2007) recording and has been used in previous studies (e.g., Dammen et al., 2015) with permission from the originator for the current study. After introduction and practice in session, the patients will be asked to perform ATT daily as a homework assignment and in-session practice of ATT will be used throughout the treatment. All patients will be encouraged to practice ATT twice a day as homework between the sessions during the first 3 weeks. Compliance and response to ATT will be recorded for each patient.

### 2.7. Wait list control

This will involve monitoring patients waiting to receive ATT for a period of 6–8 weeks. These patients may access on demand follow up consultation at general practitioners. At the end of this period, patients will be re-assessed prior to being allocated to receive group ATT.

### 2.8. Training, supervision and treatment fidelity

The therapist (TD) performing the treatment has completed Level 1 and 2 training in MCT, published studies on group metacognitive therapy in depressed patients (Dammen et al., 2015, 2016), and thus is familiar with ATT treatment. In order to maintain treatment fidelity, supervision will be provided by CP on a weekly basis. CP has received training from the originator of ATT. In addition, all sessions will be audiotaped and a random sample of 10% will be checked for therapist competence and treatment fidelity as rated by independent MCT experts using a checklist. Inter-rater Kappa coefficients for competency/fidelity ratings will be reported.

### 2.9. Data collection: participant timeline

Patients will complete measures at baseline, mid-treatment, post-treatment and 6 months follow up. Details are shown in Table 1. No financial compensation will be provided to the participants.

### 2.10. Criteria for discontinuation of participants

Participants may withdraw from the study at any time without any consequences and without giving any reasons. Participants who chose to withdraw will receive usual care. Patients can also be withdrawn at the request of the principal investigator if a participant is deemed to require other urgent treatment, e.g., hospitalization because of somatic illness or need of other specialist care.

### 2.11. Outcomes

Primary outcomes are: changes in HADS-anxiety and HADS-depression scores from pre-treatment to post-treatment and 6 months follow-up between treatment arms. Changes in the proportion of patients scoring below 8 on HADS-anxiety and HADS-depression, respectively, from pre-treatment to post-treatment and 6-months follow-up between treatment arms.

Secondary outcomes are changes in psychiatric disorders, rumination, worry, type D-personality, insomnia, quality of life and metacognitions scores, as well as CRP levels, from pre-treatment to post-treatment and 6-months follow-up between treatment arms. In addition, qualitative data will be collected and evaluated.

### 2.12. Measures

#### 2.12.1. Primary outcome

Hospital Anxiety and Depression Scale: The HADS (Zigmond and Snaith, 1983) is a 14 item self-report scale measuring anxiety (7 items) and depression (7 item). Responders rate distress based on the past 7 days. Each item is scored using a 4-point Likert-scale (from 0 to 3). A score ≥ 8 on either of the subscales indicates a significant level of anxiety and/or depression, respectively (Bjelland et al., 2002). Scores from 0 to 7 are categorized as normal, from 8 to 10 as mild, 11–14 moderate and 15 to 21 severe. The HADS has been employed in several CHD studies with good psychometric properties (Haddad et al., 2013).

#### 2.12.2. Secondary outcomes

##### 2.12.2.1. Diagnostic interviews

A structured interview for DSM-5 Disorders (SCID-5-CV) (First et al., 2016), and a structured clinical interview for DSM-V axis II disorders (SCID-5- PD) (First et al., 2015) will be used.

##### 2.12.2.2. Ruminative Response Scale (RRS)

The RRS (Nolen-Hoeksema and Morrow, 1991) is a 22-item scale assessing tendency to ruminate in response to depressed mood. The RRS contains items tapping the meaning of rumination (e.g., “I write down what I am thinking about and analyze it”), the feelings related to depressed mood (e.g., “I think about how sad I feel”), symptoms of depression (e.g., “I think about how hard it is to concentrate”), and consequences and causes of depressed mood (e.g., “I think I will not be able to do my job if I do not snap out of this”). Responses are required on a 4-point rating scale ranging from 1 (*almost never*) to 4 (*almost always*). The reported Cronbach alpha for the RRS is 0.92 (Nolen-Hoeksema and Morrow, 1991). We will apply the total score.

##### 2.12.2.3. Penn state worry questionnaire (PSWQ)

The PSWQ (Meyer et al., 1990) is a 16-item self-report questionnaire designed to measure the trait worry. Examples of items of the PSWQ are “I worry all the time” and “My worries overwhelm me.” Responses are rated on a five-point Likert scale ranging from 1 (“not at all typical of me”) to 5 (“very typical of me”). The PSWQ has good psychometric properties (Startup and Erickson, 2006). The reported Crohnbach alpha for the PSWQ is 0.93 (van Rijsoort et al., 1999).

##### 2.12.2.4. Type D personality questionnaire

The type D personality questionnaire (DS14) (Denollet, 2005) is a self-report scale consisting of two subscales, negative affectivity and social inhibition. Each subscale consists of seven items, amounting to a total of 14 items and rated on a five-point Likert scale (0–4). A person is identified as having type D personality if the score on both scales is ≥10.

##### 2.12.2.5. Bergen Insomnia Scale (BIS)

The BIS (Pallesen et al., 2008) is a six-item questionnaire based on the criteria for the clinical diagnosis of insomnia described in the *Diagnostic and Statistical Manual of Mental Disorders*, fourth version (DSM-IV-TR). The first four items inquire about difficulties with sleep initiation, maintenance of sleep, awakenings in the morning, and nonrestorative sleep; items five and six assess daytime impairment and satisfaction with sleep. A 30 min cutoff value is used for the first three items. All items are scored as number of days per week (0–7), yielding a continuous sum score from 0 to 42 (BIS sum score), increasing with severity of insomnia symptoms. The BIS can also be used as a diagnostic tool (insomnia vs. no insomnia). Three days or more on items one, two, three, or four combined with 3 days or more on item five or six indicate a diagnosis of insomnia. The BIS has adequate psychometric properties, and there are normative Norwegian data for comparison (Pallesen et al., 2008). In the present study, the 4-week test–retest reliability of the BIS was 0.92 (Peersen et al., 2017). The BIS was included in this study because BIS-insomnia has been associated with poor prognosis in CHD patients (Frøjd et al., 2022a) and our previous feasibility study demonstrated ATT to reduce insomnia (Dammen et al., 2022).

##### 2.12.2.6. Short Form−12 (SF-12)

The SF-12 (Ware et al., 1996) is a 12-item self-report questionnaire that rates on a five-point Likert scale. It assesses health outcomes from a patients’ perspective and measures health related quality of life (HRQoL). It is designed so that a higher score indicates a better health state. The questionnaire has items measuring physical functioning (two items), role-physical (two items), bodily pain (one item), general health (one item), vitality (one item), social functioning (one item), role-emotional (two items) and mental health (two items).

##### 2.12.2.7. Metacognitions Questionnaire−30 (MCQ-30)

The MCQ-30 (Wells and Cartwright-Hatton, 2004) is a 30-item self-report scale. It measures five different domains of metacognition (cognitive confidence, positive beliefs about worry, cognitive self-consciousness, negative beliefs about uncontrollability and danger, need to control thoughts). Each item is measured on a 4-point Likert scale (ranging from 1 to 4), and based on agreeableness to the specific item.

##### 2.12.2.8. Negative Beliefs about Rumination Scale (NBRS)

The NBRS (Papageorgiou et al., 2001) is a 13-item scale assessing negative metacognitive beliefs about rumination. Example items include “Ruminating about my problems is uncontrollable” and “People will reject me if I ruminate.” Respondents are required to indicate the extent to which they agree with each of the items on a 4-point rating scale ranging from 1 (*do not agree*) to 4 (*agree very much*). The reported Cronbach alpha for the NBRS is 0.82 (Papageorgiou et al., 2001).

##### 2.12.2.9. Positive Beliefs about Rumination Scale (PBRS)

The PBRS (Papageorgiou and Wells, 2001) is a 9-item scale that assesses positive metacognitive beliefs about the perceived benefits and advantages of rumination. Example items include “I need to ruminate about my problems to find answers to my depression” and “Ruminating about the past helps me to prevent future mistakes and failures.” Respondents are required to indicate the extent to which they agree with each of the items on a 4-point rating scale ranging from 1 (*do not agree*) to 4 (*agree very much*). The reported Cronbach alpha for the PBRS is 0.89 (Papageorgiou and Wells, 2001).

#### 2.12.3. Sociodemographic and clinical data

Other data from hospital medical records and self-report questionnaires collected at baseline will include gender, age, information about CHD index event and treatment, time elapsed between the index event and randomization, somatic comorbidity and risk factors (diabetes and hypertension), marital status (single, married or cohabiting, divorced, separated or widowed), education (in years), age at onset of depressive or anxiety episodes, length of depressive/anxiety illness, duration of current anxiety/depressive episode, number of previous depressive episodes, previous therapy for mental disorders and use of alcohol and medication (including sedatives/hypnotics).

#### 2.12.4. C-Reactive protein (CRP)

High sensitivity CRP will be measured at the start of the study, end-of-treatment and follow up. Non-fasting venous whole blood will be sampled in an EDTA-tube analyzed on a clinical chemistry analyzer (Architect ci16200, Abbott Laboratories, Abbott Park, Illinos, USA) at Drammen Hospital to prevent inter-laboratory bias.

#### 2.12.5. Interview guide

An interview guide was developed for the qualitative part of the study by our inter-disciplinary research group.

### 2.13. Sample size and power calculation

Statistical power calculation was based on the primary outcome of HADS with subscale outcomes of HADS-depression and HADS-anxiety. A conservative anxiety/depression effect size from previous studies of individual and group ATT for depression was used (0.67). This effect size is similar to what has been reported in a meta-analytic study of CBT and other psychological treatments (0.67) (Cuijpers et al., 2010). Thus, based on previous research, we expected a difference between the two conditions (ATT and WLC) amounting to an effect size of approximately 0.67. Allowing for 15–20% drop out in accordance with previous studies, we aim for a final sample size of 32 in each group, i.e., 64 in total. This yields a statistical power above 90% using a two-sided significance test (*p* < 0.05).

### 2.14. Analyses

The study will be analyzed using quantitative and qualitative methods.

#### 2.14.1. Quantitative analysis

A repeated measures linear mixed-model will be used to estimate treatment effects for the combined sample, with HADS-A and HADS-D scores as outcomes, from pre-treatment, post-treatment and 6 months follow-up. Controlled effect sizes will be calculated in the following way: post treatment for immediate ATT minus post waiting list divided by the pooled standard deviation. Both ITT and per protocol analyses will be reported.

The primary reported analyses will be carried out as an intention-to-treat with all randomized participants. The analysis is supplemented with per-protocol analyses defined by participation in ≥4 of the 6 group-based sessions. Missing information on other co-variates will be permitted if there is <20% missing data for the co-variate in question. Age, gender, HADS-D and HADS-A scores will be entered in the models as covariates if baseline differences are observed.

An estimate of treatment effect size will be derived by comparing the wait-list control and intervention group on levels of symptoms of anxiety and depression (HADS), rumination (RRS), worry (PSWQ), insomnia (BIS), metacognitive beliefs (MCQ-30, PBRS, NBRS) and inflammation (CRP) at baseline, end of treatment and 6 months follow-up. False discovery rate will be applied for multiple testing (Benjamini and Hochberg, 1995).

Analyses will be undertaken using Stata SE vs. 18.0 (StataCorp, 2023) and/or R (R Core Team, 2021) and conducted by a statistician who will remain blind to group allocation throughout. A detailed statistical analyze plan (SAP) will be prepared and published prior to analyses taking place.

#### 2.14.2. Qualitative analysis

Ten patients who have received ATT will be asked to participate in this qualitative study. The interviews will last for up to 60 min and will be conducted by KT. A phenomenological/hermeneutic approach will be used to analyze the data (Laverty, 2003). Thematic content analysis will be conducted to identify themes and patterns (Braun and Clarke, 2006). A semi- structured qualitative interview protocol has been developed for these purposes. This interview will cover the following themes: Experiences with ATT, experiences of processes that will be followed by improvement, experiences of aspects that might be unhelpful, and experiences with external factors (family, friends, work, school etc.) that will be important for improvement.

In-depths interviews will be analyzed by using Braun and Clarke’s 6 stage method for thematic analysis (Braun and Clarke, 2006). The transcripts will be read several times by the co-authors (KT and JM) to familiarize with the content and to form an overall impression of the material. The analysis will be deductive from the interview guide and supplemented inductively. Transcripts will be imported into the computer software NVivo 12 (QSR International Pty Ltd, 2018) a tool for analyzing qualitative data, for organizing and encoding data. Initial codes will be created based on statements of what was useful or what was not useful. The codes will be grouped into overarching themes, and sub-themes will be identified within those themes. The data analysis will be carried out by KT, in regular contact with JM and CP throughout the analysis process to discuss and redefine themes and sub-themes. Quotes from participants will been chosen to illustrate themes and sub-themes.

### 2.15. Trial management

Vestre Viken Trust is sponsor for this investigator-initiated trial which is part of the psychosocial project of the Norwegian Coronary Prevention (NORCOR) research group.^2^ The steering committee meets every 6 months to provide expert advice, supervise the overall projects, and monitor progress against milestones. A trial management group comprising the principal investigator (TD), the NORCOR principal investigator (JM), other supervisors (CP) and core personnel meet at least biweekly to monitor progress and be responsible for the day-to-day management of the project. There is also a user group with CHD patients, general practitioners, cardiac nurses, and user-representatives from patient organizations who have provided feedback on the study design and other aspects of the protocol. They will also contribute to disseminate the results and other trial related activities.

### 2.16. Data management

Data collection and coordination will be conducted by the study coordinator under the responsibility of the principal investigator.

### 2.17. Trial status and timeline

The first patient was randomized November 30th 2022 and per May 2023 10 out of 64 patients have been included and followed-up. A conservative estimate is that all 64 patients may be enrolled over a 2 year period. Thus, the final results of primary endpoint are expected in December 2024.

### 2.18. Study safety

We are not aware of any side-effects of ATT and no side-effects or serious events have been reported in previous ATT studies. However, group-ATT will be delivered by a health professional, who will monitor all study participants who attend the group sessions for any potential serious or adverse events. If any should occur during the treatment, the participants will be offered individual counseling and referral for relevant treatment elsewhere if considered indicated.

## 3. Discussion

The ATT-CHD study has three overarching purposes. First, and most importantly, the study will explore whether ATT could reduce psychological distress in outpatients with CHD. This is important because significant symptoms of anxiety and depression are common in CHD patients, also in longer periods of time after an acute cardiac event. In addition, such symptoms and their comorbidities have been associated with an up to 2–3- fold increased risk of cardiovascular events and mortality (van Melle et al., 2004; Watkins et al., 2013). In addition, anxiety and depression symptoms are associated with poorer quality of life, higher rates of hospital admissions and increased cost to the health care system (Wells et al., 2020).

Second, the ATT-CHD study will explore whether a biological marker that may link to psychological factors to cardiac prognosis could be correlated to a change in the level of psychological distress. Thereby also providing further insight into the underlying mechanisms of a changed outcome in this patient group.

The third purpose is to provide qualitative data on the patients experience with ATT including facitators and barriers to the effectiveness of treatment in order to help further improvement in effective delivery and implementation of ATT in cardiac practice.

ATT is a brief treatment that has been found to be effective in treating symptoms of depression and anxiety in mental health care settings and among student populations (Haukaas et al., 2018). Furthermore, it has been found both feasible and potentially effective in a small study in CHD patients (Dammen et al., 2022). Identifying therapeutic strategies that are both feasible, effective as well as implementable, could help reduce levels of psychological distress such as anxiety and depression in this patient group. If effective, this treatment has great potential to be implemented in outpatient care as well as in cardiac rehabilitation units. A reduction in psychological distress could lead to better psychological and cardiac outcomes, improved quality of life, and potentially be cost-effective to health care systems.

Finally, this clinical trial offers further testing and assessment of a treatment method/technique that is both brief, and with appropriate training and supervision, we believe that ATT can be competently and effectively delivered.

## Ethics statement

The studies involving humans were approved by Regional Committee of Ethics (REK) South East Norway (REK 52002); data protection officer at Drammen Hospital (20/04048-1/005). The studies were conducted in accordance with the local legislation and institutional requirements. The participants provided their written informed consent to participate in this study.

## Author contributions

TD, CP, JM, and KT conceived and designed the study and acquired data and drafted the manuscript. CP and TD designed the intervention. TD performed the intervention under supervision of CP. KT provided data and responsible for the qualitative study. OK and KT performed the statistical analysis and drafted the statistical analysis plan. All authors read and approved the final manuscript.

## Funding

This study was funded by grants from South-Eastern Norway Regional Health Authority (Helse Sør-Øst RHF/Grant number 2019125), the participating hospitals and the University of Oslo. Open Access publication was funded by the University of Oslo.

## Conflict of interest

The authors declare that the research was conducted in the absence of any commercial or financial relationships that could be construed as a potential conflict of interest.

## Publisher’s note

All claims expressed in this article are solely those of the authors and do not necessarily represent those of their affiliated organizations, or those of the publisher, the editors and the reviewers. Any product that may be evaluated in this article, or claim that may be made by its manufacturer, is not guaranteed or endorsed by the publisher.

## Footnotes

^1^https://www.sealedenvelope.com/simple-randomiser/v1/lists

^2^www.norcor.no

## Abbreviations

ATT: Attention Training Technique
CHD: Coronary Heart Disease
CR: Cardiac Rehabilitation
CRP: C-Reactive Protein
ECG: Electrocardiogram
GP: General Practitioner
HADS: Hospital Anxiety and Depression Scale
MCQ-30: Metacognitions Questionnaire-30
MCT: Metacognitve Therapy
NBRS: Negative Beliefs about Rumination Scale
PBRS: Positive Beliefs about Rumination Scale
PTSD: Post Traumatic Stress Disorder
RRS: Ruminative Response Scale
SF-12: Short Form-12
WLC: Wait-List Control
